# Toward the Identification of Novel Antimicrobial Agents: One-Pot Synthesis of Lipophilic Conjugates of *N*-Alkyl d- and l-Iminosugars

**DOI:** 10.3390/md18110572

**Published:** 2020-11-19

**Authors:** Anna Esposito, Daniele D’Alonzo, Stefano D’Errico, Eliana De Gregorio, Annalisa Guaragna

**Affiliations:** 1 Department of Chemical Sciences, University of Naples Federico II, Via Cintia, 80126 Naples, Italy; anna.esposito5@unina.it (A.E.); daniele.dalonzo@unina.it (D.D.); 2 Department of Pharmacy, University of Naples Federico II, Via Domenico Montesano, 49, 80131 Napoli, Italy; stefano.derrico@unina.it; 3 Department of Molecular Medicine and Medical Biotechnology, University of Naples Federico II, Via S. Pansini 5, 80131 Naples, Italy; eliana.degregorio@unina.it; 4 Department of Chemical, Materials and Production Engineering, University of Naples Federico II, Piazzale V. Tecchio 80, 80125 Naples, Italy

**Keywords:** lipophilic iminosugars, polymer-supported triphenyl phosphine, cholesterol, antibacterial iminosugars

## Abstract

In the effort to improve the antimicrobial activity of iminosugars, we report the synthesis of lipophilic iminosugars **10a**–**b** and **11a**–**b** based on the one-pot conjugation of both enantiomeric forms of *N*-butyldeoxynojirimycin (NBDNJ) and *N*-nonyloxypentyldeoxynojirimycin (NPDNJ) with cholesterol and a succinic acid model linker. The conjugation reaction was tuned using the established PS-TPP/I_2_/ImH activating system, which provided the desired compounds in high yields (94–96%) by a one-pot procedure. The substantial increase in the lipophilicity of **10a**–**b** and **11a**–**b** is supposed to improve internalization within the bacterial cell, thereby potentially leading to enhanced antimicrobial properties. However, assays are currently hampered by solubility problems; therefore, alternative administration strategies will need to be devised.

## 1. Introduction

**Figure 1:**
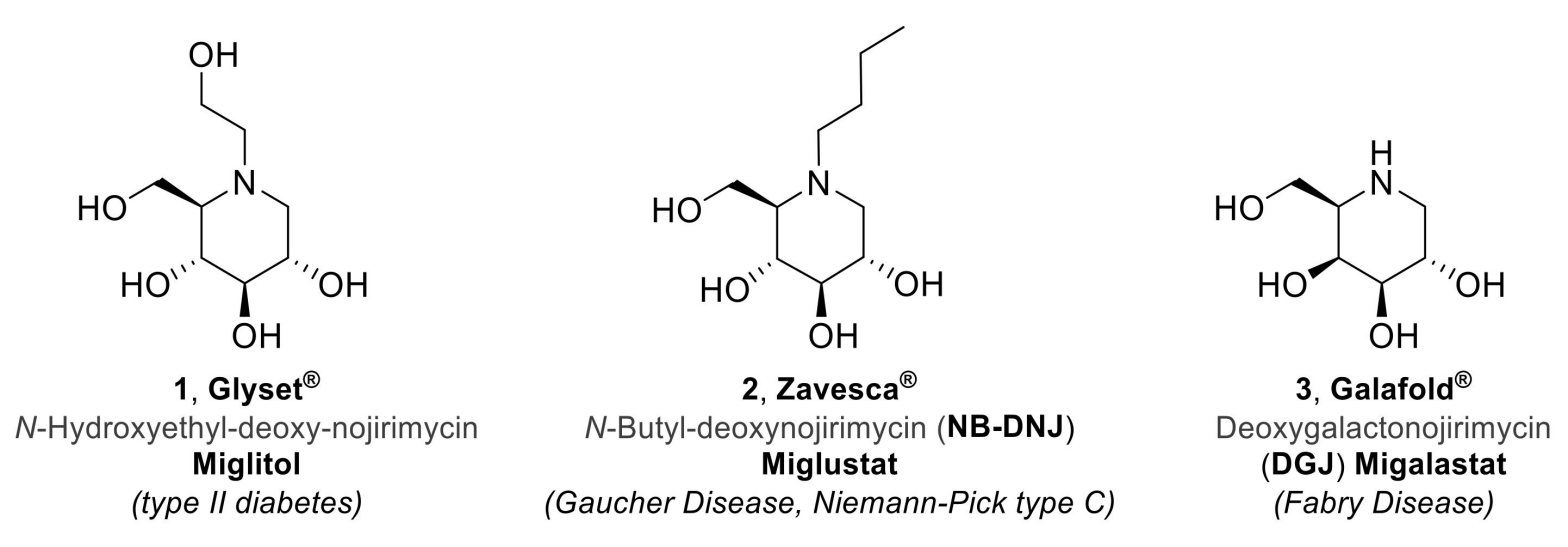
Marketed iminosugar drugs.

Iminosugars are naturally occurring or synthetic glycomimetics with an amino function replacing the endocyclic oxygen of the corresponding carbohydrates [1]. Thanks to their excellent ability to act as modulators (inhibitors/enhancers) of the activity of carbohydrate processing enzymes, including glycosyl hydrolases [2,3], glycosyltransferases [4] or glycogen phosphorylases [5], iminosugars can be considered as the main and best-settled class of sugar mimetics described so far. Over the last few decades, interesting broad-spectrum therapeutic applications have been found that have led to the development of three iminosugar drugs (Figure 1), namely Glyset (*N*-hydroxyethyl-deoxynojirimycin, Miglitol, **1**), which is used for the therapeutic treatment of type 2 diabetes [6], Zavesca (*N*-butyldeoxynojirimycin, NBDNJ, **2**), which is used for lysosomal storage disorders including Gaucher and Niemann–Pick type C diseases [7,8,9], and Galafold (deoxygalactonojirimycin, DGJ, **3**), which was recently approved for the treatment of Fabry disease [10,11]. A variety of other iminosugars have also been identified as therapeutic candidates against malignancies [12], viral infections [13,14] and other genetic disorders, including cystic fibrosis [15,16].

**Figure 2:**
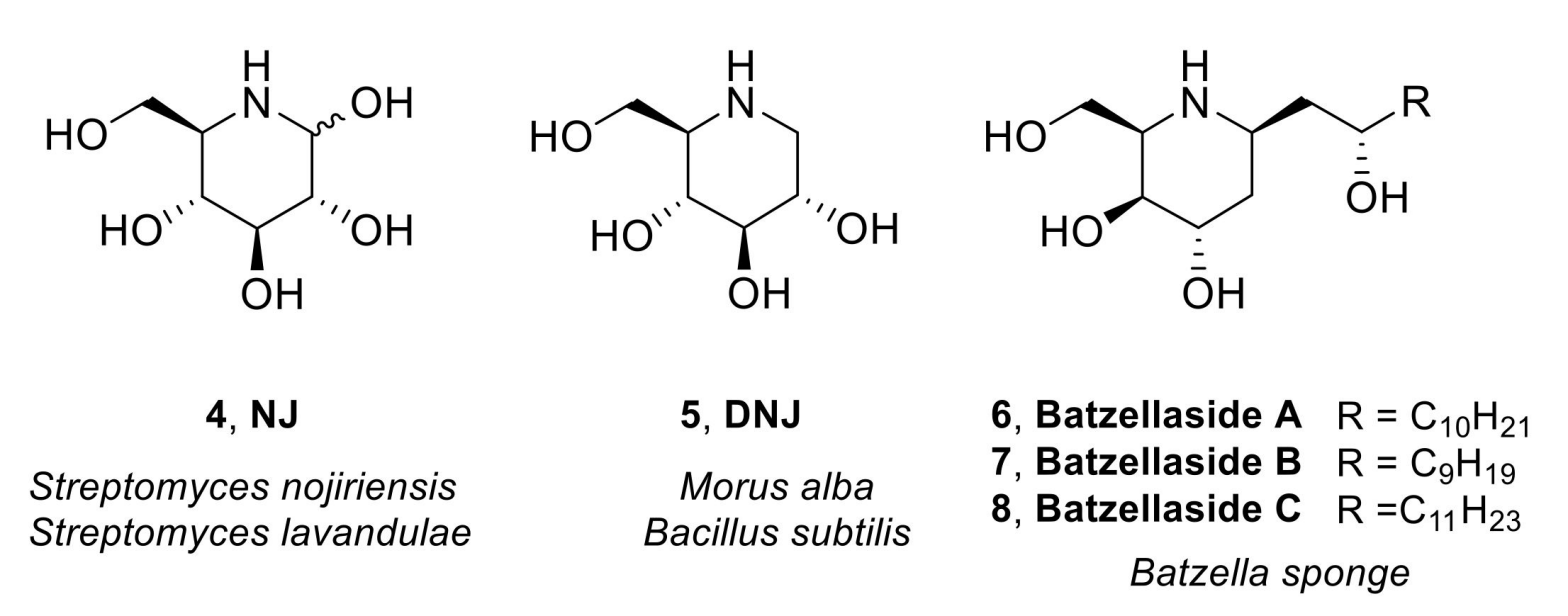
Naturally occurring iminosugars with antimicrobial properties.

However, only rarely has this class of glycomimetics been considered for its antimicrobial activity. The antibacterial effect of iminosugars was first observed to be exhibited by the iminosugar progenitor nojirimycin (NJ, **4** Figure 2) against *Xanthomanos oryzae*, *Shigella flexeneri* and *Mycobacterium* 607 [17]. Similarly, deoxynojirimycin (DNJ, **5**) was found to inhibit biofilm formation in *Streptococcus mutans* [18,19]. The antimicrobial activity against *Staphylococcus epidermidis* was also highlighted by iminosugars extracted from a marine organism, such as batzellaside A–C (**6**–**8**, Figure 2), which were isolated from a Madagascar *Batzella* sponge [20,21] (Figure 2).

**Figure 3:**
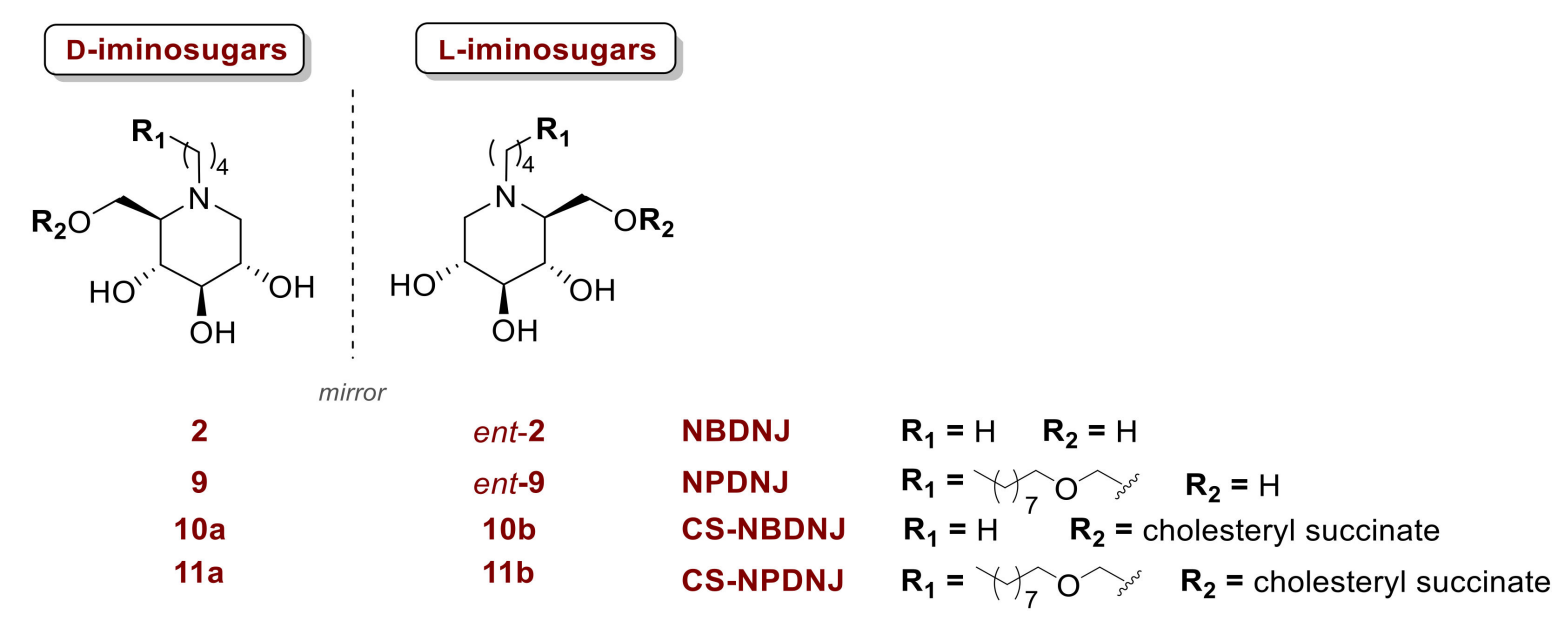
*N*-alkyl d- and l-iminosugars as antimicrobial agents against *S. aureus.*

Recently, as part of our longstanding research program that focused on the analysis of the effect of sugar chirality in the biological properties of iminosugars [15,22,23,24] and other enantiomeric bioactive compounds [25,26,27,28], we evaluated the antimicrobial potential of d- and l-DNJ, as well as that of a small library of their *N*-alkyl derivatives, against *Staphylococcus aureus* ATCC 29,213 [29]. Our data recognize a role of the lipophilicity of iminosugars in contributing to their antibacterial activity against *S. aureus* more evidently for the l-enantiomers. This led to the identification of *N*-nonyloxypentyl-l-DNJ (l-NPDNJ, *ent*-**9** Figure 3) as an interesting candidate, because of its capacity to affect growth, biofilm formation and virulence factor expression of *S. aureus* [29].

Based on the assumption that the role of lipophilicity in the antimicrobial activity of *N*-alkyl iminosugars is mainly due to the favorable internalization of the molecules increasing along with the *N*-alkylation degree, we suggested that the conjugation of iminosugars with lipophilic moieties (even at different positions than the amino group) through a cleavable linker would positively contribute to iminosugar delivery and release within the bacterial cell. Accordingly, we conceived the preparation of iminosugars **10** and **11** bearing a cholesterol unit as the lipophilic group jointed by a short succinic bis-ester, as model linker [30,31], to the iminosugar moiety. In these early synthetic efforts, we selected as iminosugars *N*-butyl d- and l-DNJ (d- and l-NBDN, **2** and *ent*-**2** Figure 3) and *N*-nonyloxypentyl d- and l-DNJ (d- and l-NPDNJ, **9** and *ent*-**9** Figure 3). Contrary to the aforementioned activity of the latter, the former did not display antimicrobial properties against *S. aureus* ATCC 29,213 [29]. In line with our assumption, we studied whether conjugation could confer antibacterial activity to otherwise ineffective *N*-alkyl iminosugars while improving the properties of those already bearing an antimicrobial potential.

## 2. Results and Discussion

### Chemistry

**Scheme 1:**
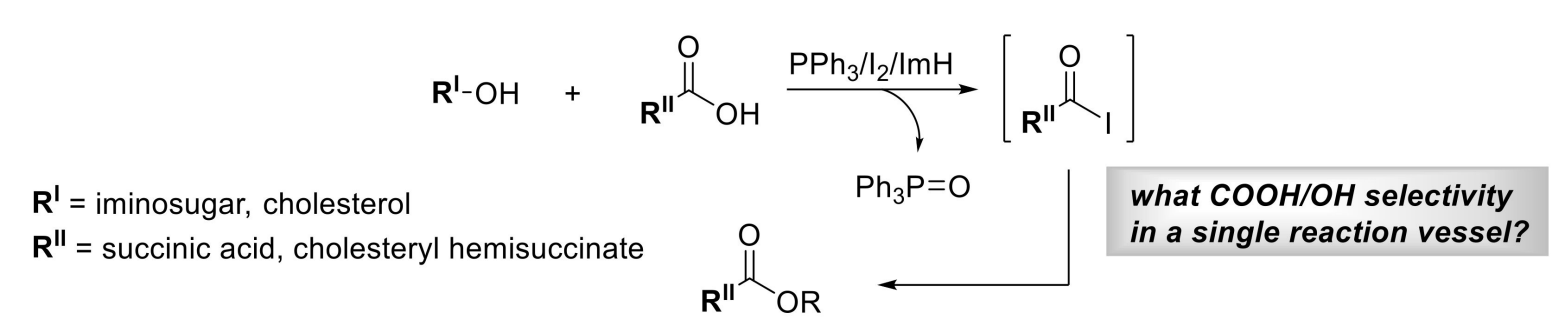
Selectivity (COOH vs. OH) in the TPP/I_2_/ImH reaction.

Our synthetic strategy was aimed to afford iminosugar conjugates **10a**–**b** and **11a**–**b** from the corresponding starting materials in a single reaction vessel, using a method that was able to activate the carboxylic moieties of succinic acid while leaving the remaining functionalities of cholesterol and iminosugars unreacted. To this end, we conceived as an activating agent the system based on the combined use of triphenyl phosphine (TPP) or its polymer-supported variant (PS-TPP), molecular iodine (I_2_) and imidazole (ImH). The PS-TPP/I_2_/ImH system has long been at the core of our synthetic studies aimed at many transformations, including the synthesis of β-amino acids [32] and of deuterated fatty acids [33,34], the formation of glycosyl iodides [35], the phosphorylation of nucleosides [36], the acetalization of sugars [37] and steroids [38] and the one-pot alkylation of iminosugars [15]. In all cases, the activation of alcohol or carboxylic acid functions to the corresponding iodides was achieved by the reaction of the last ones with pre-formed triphenyl-di-imidazolyl phosphorane [39] and congeners thereof, ultimately releasing the iodinating product and triphenylphosphine oxide. The use of TPP/I_2_/ImH in one-pot procedures has already been successfully demonstrated [15]. In this case, a still unanswered question regards the selectivity of carboxylic acids vs. alcohols (Scheme 1), if both nucleophiles occur concurrently when performing the reaction in a single vessel. 

**Scheme 2:**
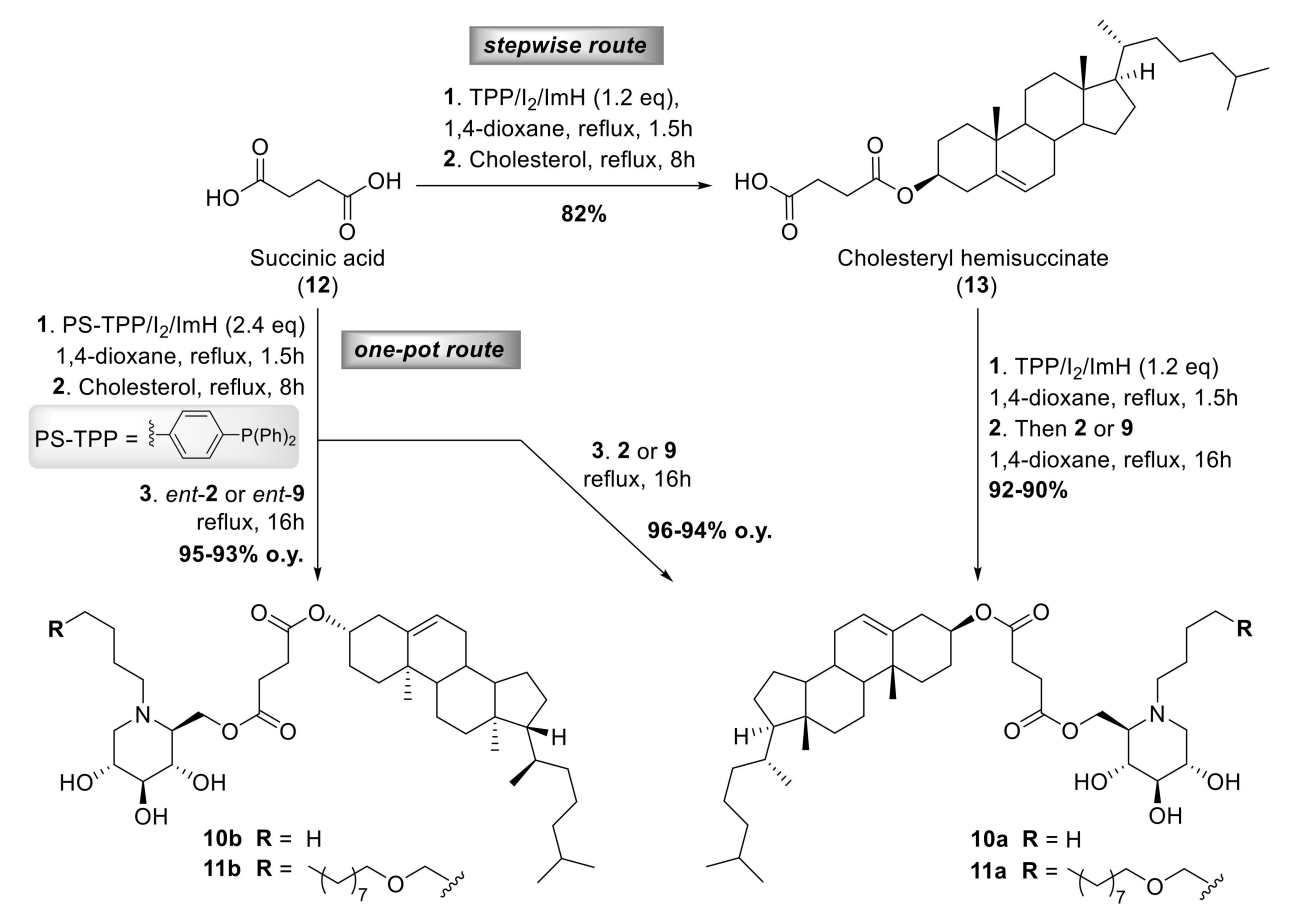
Stepwise and one-pot routes to iminosugar conjugates **10a**–**b** and **11a**–**b**.

A stepwise procedure was initially explored to optimize the single reaction conditions. First, we studied the coupling reaction of succinic acid with cholesterol (Scheme 2). Succinic acid (**12**) was first treated with the pre-formed TPP/I_2_/ImH complex in refluxing dioxane for 1.5 h (dioxane has been identified as the most suitable solvent among all those tested, mostly considering the best solubility of all reagents in view of the one step procedure). Then, upon cholesterol addition, the reaction mixture was left while stirring under reflux for 8 h, yielding cholesteryl hemisuccinate (**13**) in 82% (Scheme 2). The reaction gave roughly the same results when cholesterol was reacted, using a previously described procedure [40] with succinic anhydride and triethylamine in hot toluene (60 °C) for 8 h. 

The subsequent coupling reaction of hemisuccinate **13** with unprotected iminosugars **2**,**9** and *ent*-**2**,**9** was then studied (Scheme 2). Successful activation of **13** with premixed TPP/I_2_/ImH (1.2 eq) under previously described conditions (refluxing dioxane, 1.5 h) was suggested by the formation of a low-polarity, UV–visible [41] spot in the TLC analysis of the reaction mixture, which was a hint of covalent iodine incorporation by the starting material. The subsequent addition of d-NBDNJ (as model substrate) provided the desired cholesterol–iminosugar conjugate **10a** (stepwise route, Scheme 2). The complete conversion of the reagent (up to 92% yield) was achieved when heating the reaction mixture at reflux for 16 h. NMR analysis confirmed the occurred conjugation at the C6-OH group of d-NBDNJ, as indicated by the shift of diastereotopic methylene protons of the iminosugar moiety in DMSO-*d*_6_ (**2**: H-6a, 3.54 ppm, H-6b, 3.72 ppm; **10a**: H-6a, 4.02 ppm, H-6b, 4.37 ppm). It is worth noting that more established coupling conditions were found as not efficient in this case. As an example, the reaction of hemisuccinate **13** and d-NBDNJ (**2**) with 2-(1H-benzotriazole-1-yl)-1,1,3,3-tetramethylaminium tetrafluoroborate (TBTU) and DIPEA, which has already been shown to succeed in the esterification of similar substrates [42], furnished conjugate **10a** only in moderate yield (49%; see Scheme S1 Supplementary Materials).

The above reactions pointed out the efficacy of the TPP/I_2_/ImH system as coupling reagent for esterification reactions. Moreover, they indicated full compatibility and selectivity between alcohols and carboxylic acids of the reagents. However, it should be noted that only a slight excess of the iodinating complex (1.2 eq/eq of COOH) was always added to avoid further iodination reactions. The success of the stepwise approach enabled us to repeat the reaction using a one-pot procedure (Scheme 2). In this case, we chose to replace TPP with PS-TPP and to increase the synthetic potential of the procedure on a higher scale [15]. Accordingly, succinic acid was added to a stirring suspension of a 2-fold amount of PS-TPP/I_2_/ImH (2.4 eq) to ensure the activation of both COOH functions for conjugation to both cholesterol and the iminosugar. After the addition of cholesterol, d-NBDNJ was added to the newly formed cholesteryl hemisuccinate iodide. In this case, the use of PS-TPP did not alter the overall reactivity; therefore, the reaction conditions tuned up with soluble TPP could be simply repeated. Filtration of the crude removed the residual phosphine and its oxide, while extractive work-up was required to remove the excess of imidazole. Eventually, the desired conjugate **10a** was recovered in a satisfying 96% yield. The same conditions were also effective when l-NBDNJ [24] was used as the iminosugar, leading to conjugate **10b** in 95% yield. Similarly, the reaction with d- and l-NPDNJ provided the corresponding conjugates **11a** and **11b** in 90% and 93% yields, respectively. As expected, ^1^H NMR analysis of diastereomeric couples **10a**–**10b** and **11a**–**11b** indicated that the corresponding signals have wholly superimposable chemical shifts and multiplicities, presumably owing to the lack of interactions between the chiral centers of cholesterol and those of iminosugar enantiomers.

Preliminary biological evaluation of **10a**–**b** and **11a**–**b** was performed by broth microdilution assays, with the aim to measure the minimum inhibitory concentration (MIC) of iminosugar conjugates against *S*. *aureus*, a human pathogen that is responsible for a wide range of hospital-associated infections and has the capacity to develop multi-resistance to antibiotics. However, the assays were strongly hampered by the very limited solubility of the conjugates in the bacterial culture broth, which always precipitated even after pre-solubilization in DMSO. Studies are currently ongoing to overcome these limitations by searching for alternative solutions that will exploit the amphiphilic character of the glycomimetic agents.

## 3. Materials and Methods 

### 3.1. Chemistry

All commercially available reagents and solvents were purchased at the highest degree of purity from commercial sources and used without purification. TLC analysis was carried out on precoated silica gel plate F254 (Merck), and products were visualized under UV radiation or by exposure to iodine vapor and chromic mixture. Column chromatography was performed with silica gel (70–230 mesh, Merck Kiesegel 60). CHNS analysis was performed to assess the purity of compounds and was ≥95% in all cases. NMR spectra were recorded on a Bruker AVANCE 400 MHz. Coupling constant values (*J*) were reported in Hz. Chemical synthesis and structural characterization of d-and l-NBDNJ (**2** and *ent*-**2**) and d-and l-NPDNJ (**9** and *ent*-**9**) were achieved as previously reported [15,24].

#### 3.1.1. Procedure for the Synthesis of **10**–**11** through a Stepwise Route

##### Step 1: preparation of cholesteryl hemisuccinate **13**

I_2_ (1.2 eq) and imidazole (2.4 eq) were added to a stirred solution of TPP (1.2 eq) in anhydrous 1,4-dioxane ([I_2_] = 62.5 mM) at rt. After 10 min, succinic acid (1.0 eq) was added to the slight yellow suspension, and the pH of the solution was adjusted to neutrality with the addition of imidazole. The resulting colorless solution was warmed to reflux temperature and stirred for 1.5 h. Cholesterol (1.0 eq) was then added, and the mixture was stirred at the same temperature for 8 h. The mixture was then cooled at rt, washed with brine and extracted with DCM. Organic layers were dried (Na_2_SO_4_) and evaporated under reduced pressure. Column chromatography of the crude residue over silica gel (hexane/EtOAc = 6:4) afforded the pure **13** (82% yield). ^1^H and ^13^C NMR spectra were fully in agreement with those reported in the literature [40,41].

##### Step 2: preparation of iminosugar conjugate **10a**

I_2_ (1.2 eq) and imidazole (2.4 eq) were added to a stirred solution of TPP (1.2 eq) in anhydrous 1,4-dioxane at rt. After 10 min, hemisuccinate **13** (1.0 eq) was added to the slight yellow suspension, and the pH of the solution was adjusted to neutrality with the addition of imidazole. The resulting colorless solution was warmed to reflux temperature and stirred for 1.5 h. d-NBDNJ (1.0 eq) was then added, and the mixture was stirred at the same temperature for 8 h. The mixture was cooled at rt and diluted with DCM, and the organic layers were washed with brine, dried (Na_2_SO_4_) and concentrated under reduced pressure. Chromatography of the crude residue over silica gel (DCM/MeOH = 96:4) afforded the pure **10a** (92% yield) as a white solid.

#### 3.1.2. General Procedure for the Synthesis of **10a**–**b** and **11a**–**b** through a One-Pot Route

I_2_ (2.4 eq) and imidazole (4.8 eq) were added to a stirring solution of polymer-supported triphenylphosphine (PS-TPP; 100–200 mesh, extent of labeling: ~3 mmol/g triphenylphosphine loading) (2.4 eq) in anhydrous 1,4-dioxane at rt. After 10 min, succinic acid (1.0 eq) was added to the slight yellow suspension, and the pH of the solution was adjusted to neutrality by the addition of imidazole. The resulting colorless solution was warmed to reflux temperature and stirred for 1.5 h. Cholesterol (1.0 eq) was then added, and the mixture was stirred at the same temperature for 8 h. The appropriate iminosugar **2**,**9** and *ent*-**2**,**9** (1.0 eq) was then added, and the mixture was stirred for 16 h at the reflux temperature. The mixture was then cooled to rt, filtered (DCM) to remove triphenylphosphine oxide, washed with brine and extracted with DCM. Organic layers were dried (Na_2_SO_4_) and evaporated under reduced pressure, affording desired NBDNJ derivatives **10a** and **10b** (**10a**: 96% o.y.; **10b**: 95% o.y.) and NPDNJ derivatives **11a** and **11b** (**11a**: 94% o.y.; **11b**: 93% o.y.).

**10a:**^1^H NMR (400 MHz, DMSO-d_6_): δ 0.66 (s, 3H), 0.83–0.94 (m, 13H), 0.98 (s, 3H), 0.98–1.44 (m, 18H), 1.45–1.61 (m, 5H), 1.73–1.87 (m, 3H), 1.88–2.01 (m, 3H), 2.16 (ddd, *J* = 1.6, 4.3, 9.1, 1H, H-5′), 2.26 (td, *J* = 7.7, 1H, H-4), 2.25–2.36 (m, 1H, H-1″a), 2.53 (s, 4H, CH_2_CH_2_), 2.54–2.65 (m, 1H, H-1″b), 2.81 (dd, *J* = 4.8, 11.1, 1H, H-1′b), 2.92 (t, *J* = 9.1, 1H, H-3′), 3.01 (d, *J* = 5.3, 9.1, 1H, H-4′), 3.17–3.26 (m, 1H, H-2′), 4.05 (dd, *J* = 4.3, 12.0, 1H H-6′a), 4.39 (dd, *J* = 1.6, 12.0, 1H, H-6′b), 4.40–4.49 (m, 1H, H-3), 4.72 (d, *J* = 4.6, 1H, OH), 4.81 (bs, 1H, OH), 4.89 (d, *J* = 5.3, 1H, OH), 5.35 (d, *J* = 3.7, 1H, H-6). ^13^C NMR (100 MHz, DMSO-d_6_): δ 12.1, 14.4, 19.0, 19.4, 20.5, 21.0, 22.9, 23.1, 23.5, 24.2, 26.8, 27.8, 28.2, 29.2, 31.8; 35.6, 36.1, 36.5, 36.9, 38.1, 42.3, 49.9, 52.2, 56.0, 56.6, 57.2, 62.8, 64.3, 69.8, 70.9, 73.9, 79.4, 122.6, 139.9, 171.7, 172.3. [α]^25^_D_ +27.0 (*c* 1.1, DMSO). Anal. calcd for C_41_H_69_NO_7_: C, 71.58; H, 10.11; N, 2.04; O, 16.28. Found: C, 71.67; H, 10.07; N, 2.04.

**10b**: ^1^H NMR (400 MHz, DMSO-d_6_): δ 0.65 (s, 3H), 0.83–0.95 (m, 13H), 0.98 (s, 3H), 0.98–1.45 (m, 18H), 1.45–1.62 (m, 5H), 1.71–1.87 (m, 3H), 1.88–2.02 (m, 3H), 2.15 (d, *J* = 8.8, 1H, H-5′), 2.21–2.35 (m, 2H, H-4, H-1″a), 2.46 (s, 4H, CH_2_CH_2_), 2.54–2.65 (m, 1H, H-1″b), 2.81 (dd, *J* = 4.7, 11.0, 1H, H-1′b), 2.92 (t, *J* = 8.8, 1H, H-3′), 3.00 (t, *J* = 8.8, 1H, H-4′), 3.16–3.27 (m, 1H, H-2′), 4.04 (dd, *J* = 3.5, 12.0, 1H, H-6′a), 4.39 (d, *J* = 12.0, 1H, H-6′b), 4.42–4.52 (m, 1H, H-3), 4.65–4.94 (m, 3H, OH), 5.34 (d, *J* = 3.5, 1H, H-6). ^13^C NMR data for compound **10b** were superimposable with those reported above for the corresponding diastereoisomer **10a**. [α]^25^_D_ +35.0 (c 1.2, DMSO). Anal. calcd for C_41_H_69_NO_7_: C, 71.58; H, 10.11; N, 2.04; O, 16.28. Found: C, 71.52;.H, 10.14; N, 2.04.

**11a:**^1^H NMR (400 MHz, CD_3_OD): δ 0.74 (s, 3H), 0.87–0.99 (m, 12H), 1.01–1.10 (m, 5H), 1.11–1.25 (m, 6H), 1.26–1.46 (m, 19H), 1.48–1.70 (m, 12H), 1.82–2.11 (m, 5H), 2.18 (t, *J* = 11.0, 1H, H-1′a), 2.28–2.39 (m, 3H), 2.44–2.54 (m, 1H, H-1″a), 2.59–2.67 (m, 4H, CH_2_CH_2_), 2.70–2.80 (m, 1H, H-1″a), 3.03 (dd, *J* = 4.9, 11.0, 1H, H-1b), 3.15 (t, *J* = 9.0, 1H, H-3′), 3.25–3.33 (m, 1H, H-4′), 3.40–3.45 (m, 4H), 3.46–3.54 (m, 1H, H-2′), 4.29 (dd, *J* = 3.5, 12.3, 1H, H-6′a), 4.51 (dd, *J* = 1.8, 12.3, 1H, H-6′b), 5.41 (d, *J* = 4.5, 1H, H-6). ^13^C NMR (100 MHz, CD_3_OD); δ 12.3, 14.5, 19.2, 19.7, 22.2, 22.9, 23.2, 23.8, 24.9, 25.2, 25.3, 27.3, 28.8, 29.1, 29.3, 29.9, 30.3, 30.7, 30.8, 33.0; 33.2, 37.1, 37.4, 37.8, 38.3, 39.2, 40.7, 41.1, 43.5, 51.6, 53.8, 57.6, 57.7, 58.1, 62.6, 65.4, 70.8, 71.9, 72.0, 72.1, 75.7, 80.4, 123.7, 141.0, 173.4, 174.1. [α]^25^_D_ +46 (c 1.0, CH_3_OH). Anal. calcd for C_51_H_89_NO_8_: C, 72.55; H, 10.63; N, 1.66; O, 15.16. Found: C, 72.65; H, 10.59; N, 1.66.

**11b:**^1^H NMR (400 MHz, CD_3_OD): δ 0.75 (s, 3H), 0.87–1.00 (m, 12H), 1.01–1.10 (m, 5H), 1.11–1.25 (m, 6H), 1.26–1.47 (m, 19H), 1.48–1.70 (m, 12H), 1.83–2.10 (m, 5H), 2.18 (t, *J* = 10.9, 1H, H-1′a), 2.28–2.39 (m, 3H), 2.44–2.54 (m, 1H, H-1″a), 2.57–2.70 (m, 4H, CH_2_CH_2_), 2.70–2.81 (m, 1H, H-1″a), 3.03 (dd, *J* = 4.8, 11.3, 1H, H-1b), 3.15 (t, *J* = 9.0, 1H, H-3′), 3.25–3.33 (m, 1H, H-4′), 3.40–3.54 (m, 5H), 4.29 (dd, *J* = 3.4, 12.4, 1H, H-6′a), 4.52 (dd, *J* = 1.8, 12.4, 1H, H-6′b), 5.41 (d, *J* = 5.0, 1H, H-6). ^13^C NMR data for compound **11b** were superimposable with those reported above for the corresponding diastereoisomer **11a**. [α]^25^_D_ +40 (c 1.3, CH_3_OH). Anal. calcd for C_51_H_89_NO_8_: C, 72.55; H, 10.63; N, 1.66; O, 15.16. Found: C, 72.64; H, 10.60; N, 1.66.

### 3.2. Evaluation of Antibacterial Activity In Vitro

Broth microdilution assay was performed to determine the MIC value of compounds **10a**–**b** and **11a**–**b** as previously described [38]. Briefly, fresh overnight culture of *S. aureus* strains was diluted in Mueller–Hinton broth to 1 × 10^6^ colony forming units per mL (CFU/mL). One hundred microliters of bacteria suspension (1 × 10^5^ cfu) was dispensed into a 96-well microtiter plate containing the same volume of 2-fold serial dilutions of compounds **10a**–**b** and **11a**–**b**. The antibiotics gentamicin and oxacillin were used as positive controls. Following 16–24 h incubation at 37 °C, the optical density of each well was measured at 595 nm. The MICs were the lowest concentrations of compound to inhibit bacterial growth after incubation.

## 4. Conclusions

The synthesis of lipophilic iminosugars **10a**–**b** and **11a**–**b**, obtained by conjugation of NBDNJ and NPDNJ in both enantiomeric forms with cholesterol through a succinic acid linker, has been herein reported, using a one-pot procedure involving the use of PS-DPP/I_2_/ImH as an activating system. Iminosugar conjugates **10a**–**b** and **11a**–**b** have been conceived to improve internalization within the bacterial cell compared to the corresponding unconjugated *N*-alkyl iminosugars **2** and **9**; thereby, they were expected to display more favorable antimicrobial properties. However, the marked increase in the lipophilicity of the synthesized iminosugars hampered the ability to perform in vitro assays because of the very limited solubility in water. To overcome these limitations, the focus in the future will be on the development of alternative strategies for in vitro assays, eventually exploiting the amphiphilic character of the glycomimetic agents.

## Supplementary Materials

The following are available online at https://www.mdpi.com/1660-3397/18/11/572/s1. Scheme S1: Chemical synthesis of compound **10a** by an established procedure. Figure S1: ^1^H spectrum of compound **13**; Figure S2: ^1^H spectrum of compound **2**; Figure S3: ^1^H and ^13^C spectra of compound **10a**; Figure S4: ^1^H and ^1^H-^1^H COSY spectra of compound **10b**; Figure S5: ^1^H and ^13^C spectra of compound **11a**; Figure S6: ^1^H and ^1^H-^1^H COSY spectra of compound **11b**.
