# Supplementary material for: Toward the Identification of Novel Antimicrobial Agents: One-Pot Synthesis of Lipophilic Conjugates of N-Alkyl d- and l-Iminosugars
Source: Mar Drugs. 2020 Nov 19;18(11):572. doi: 10.3390/md18110572 (PMC7699595; doi:10.3390/md18110572)

SUPPORTING INFORMATION

Toward the Identification of Novel Antimicrobial Agents: One-pot Synthesis of Lipophilic Conjugates of N-Alkyl D- and L-Iminosugars

Anna Esposito[1], Daniele D’Alonzo[1], Stefano D’Errico[2], Eliana De Gregorio[3] and Annalisa Guaragna[4,*]

[1]Department of Chemical Sciences, University of Naples Federico II, Via Cintia, 80126 Naples, Italy; anna.esposito5@unina.it; daniele.dalonzo@unina.it

[2]Department of Pharmacy, University of Naples Federico II, via Domenico Montesano, 49, 80131 Napoli, Italy; stefano.derrico@unina.itstefano.derrico@unina.it

[3]Department of Molecular Medicine and Medical Biotechnology, University of Naples Federico II,
Via S. Pansini 5, 80131, Naples, Italy; eliana.degregorio@unina.it

[4]Department of Chemical, Materials and Production Engineering, University of Naples Federico II, Piazzale V. Tecchio 80, 80125 Naples, Italy, annalisa.guaragna@unina.it

* Correspondence: annalisa.guaragna@unina.it

TABLE OF CONTENTS

CHEMICAL SYNTHESIS S2

COPIES OF NMR SPECTRA S3

CHEMICAL SYNTHESIS

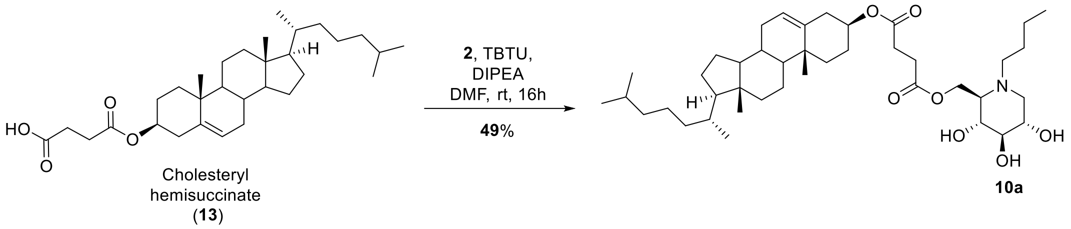

SCHEME S1. Synthesis of conjugate **10a** by established procedure

Compound 10a. Cholesteryl hemisuccinate (**13**; 0.14 g, 0.28 mmol), TBTU (9.0 mg, 0.28 mmol) and DIPEA (59 µL, 0.34 mmol) were dissolved in anhydrous DMF (1.5 mL) under nitrogen atmosphere and the resulting mixture was stirred at rt for 30 minutes. NBDNJ (50 mg, 0.23 mmol) was then added and the mixture was stirred at the same temperature for 16 h. The reaction mixture was diluted with DCM, washed with 5% HCl (2x1mL) and then with $NaHCO_3$ and water. Organic layers were dried (Na_2SO_4) and evaporated under reduced pressure. Column chromatography of the crude residue over silica gel (DCM/MeOH = 96:4) afforded the conjugate **10a** (78 mg, 49% yield). ^{1}H and ^{13}C NMR were spectra are reported below.

COPIES OF ^{1}H, ^{13}C AND ^{1}H-^{1}H COSY NMR SPECTRA

FIGURE S1. ^{1}H NMR spectrum of compound **13.**

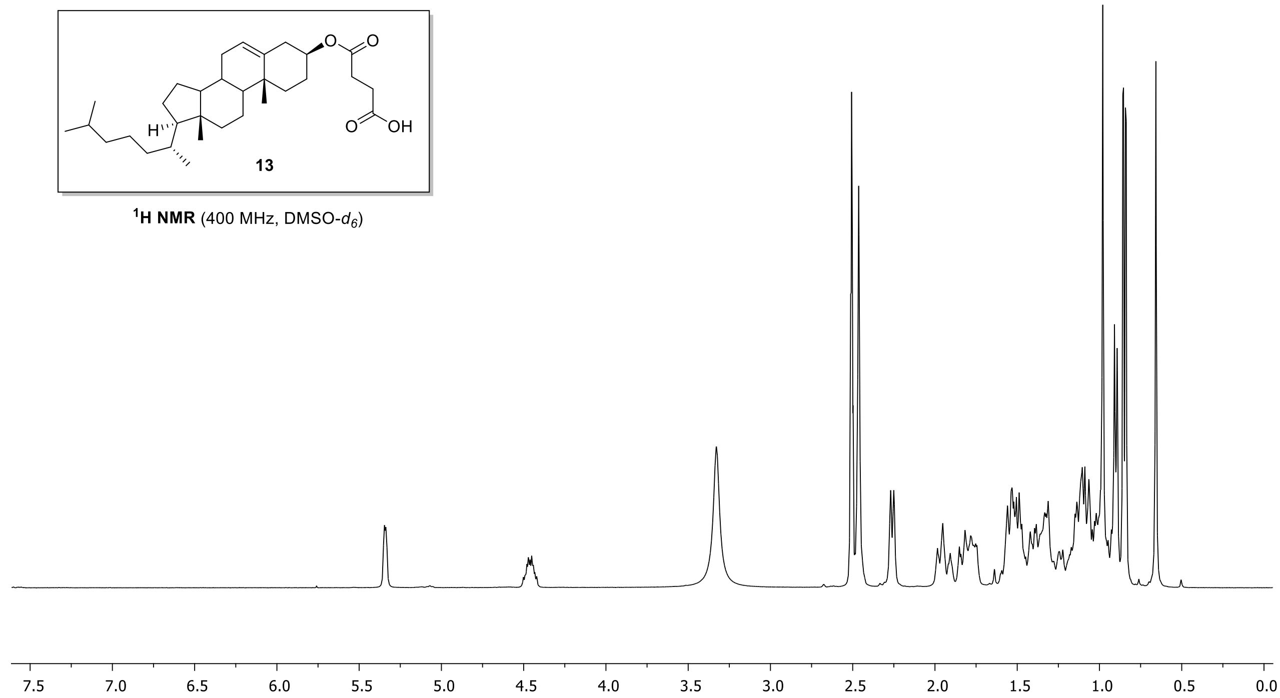

FIGURE S2. ^{1}H NMR spectrum of compound **2.**

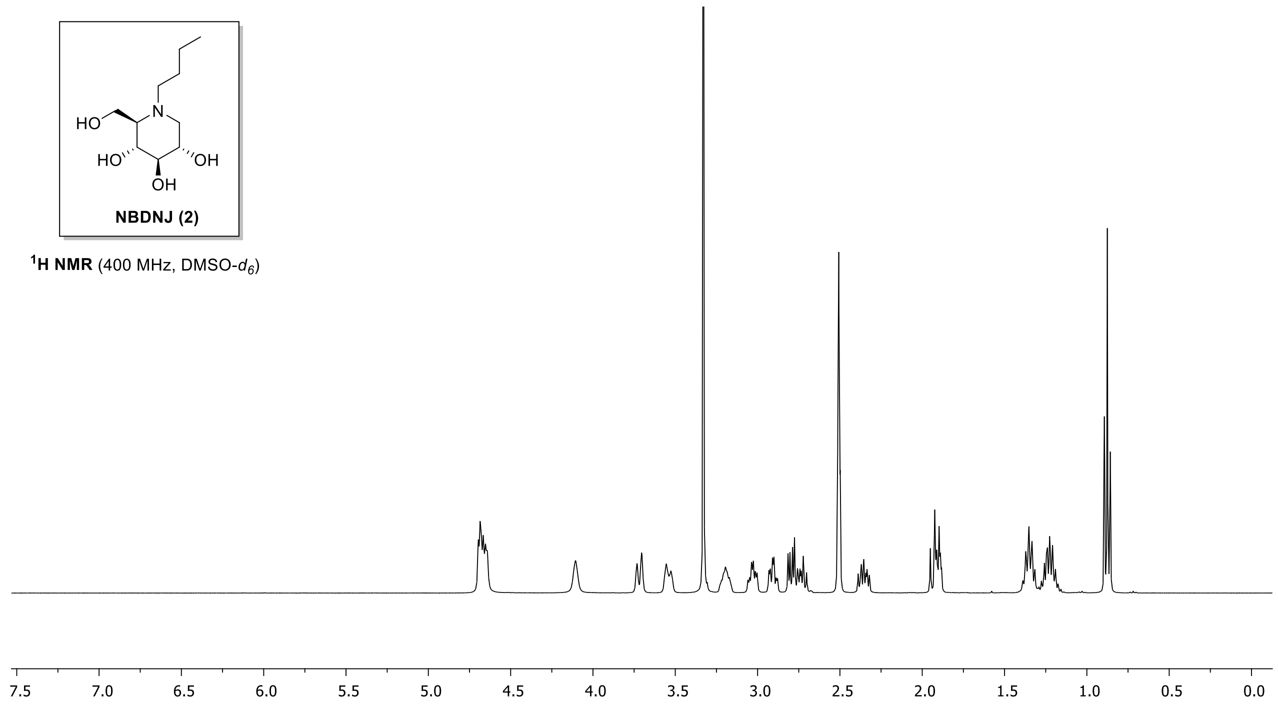

FIGURE S3. ^{1}H and ^{13}C NMR spectra of compound **10a**.

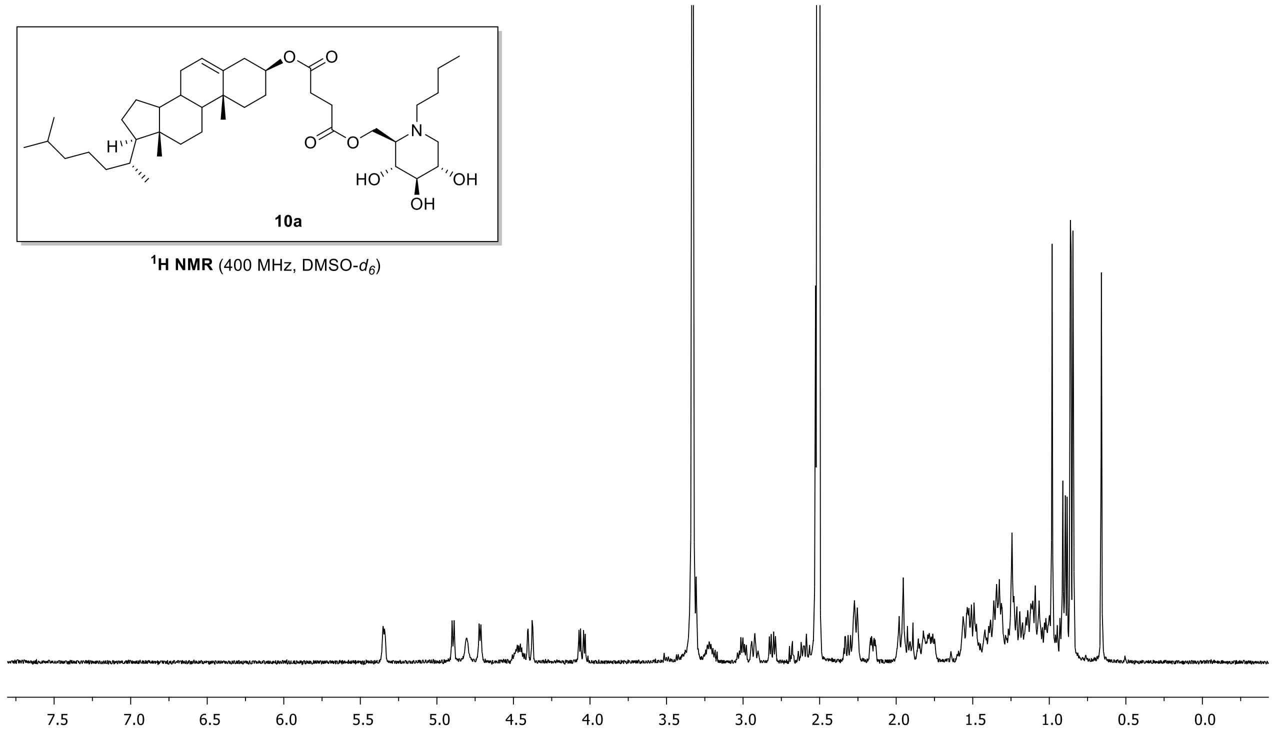

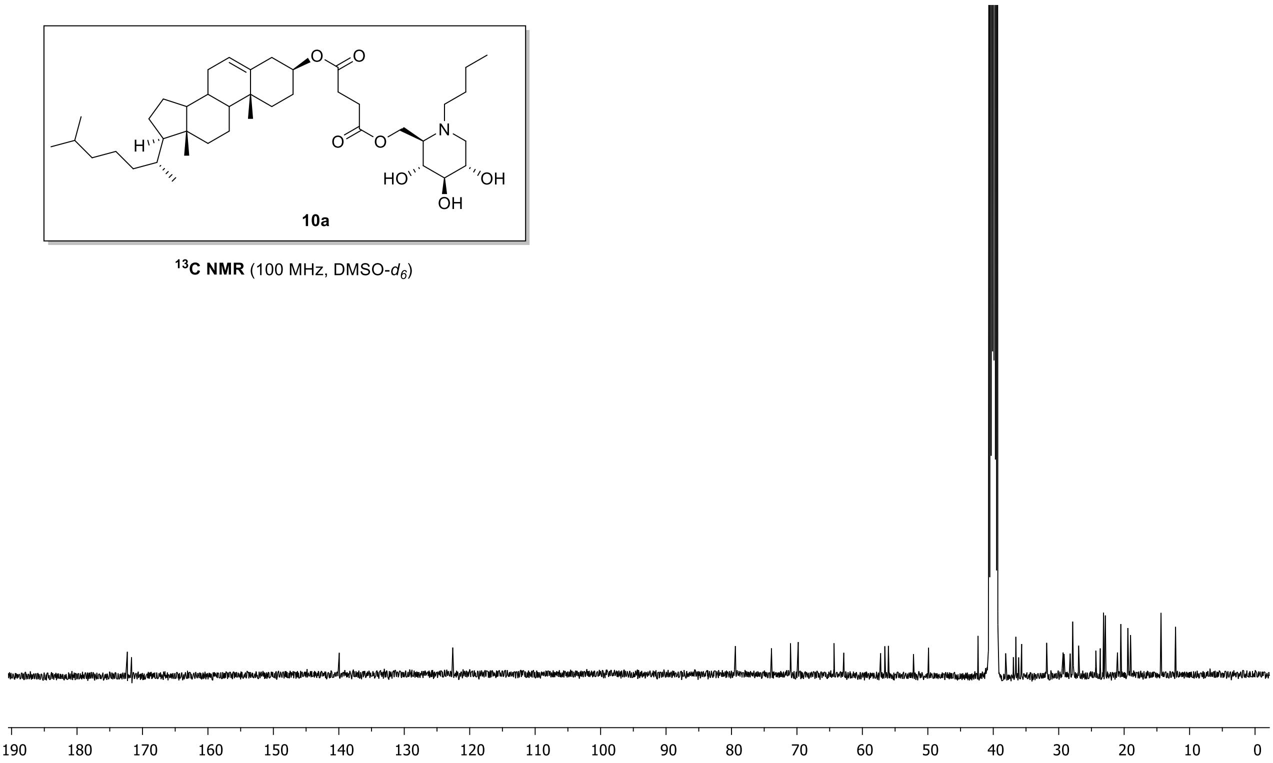

FIGURE S4. ^{1}H and ^{1}H-^{1}H COSY NMR spectra of compound **10b**.

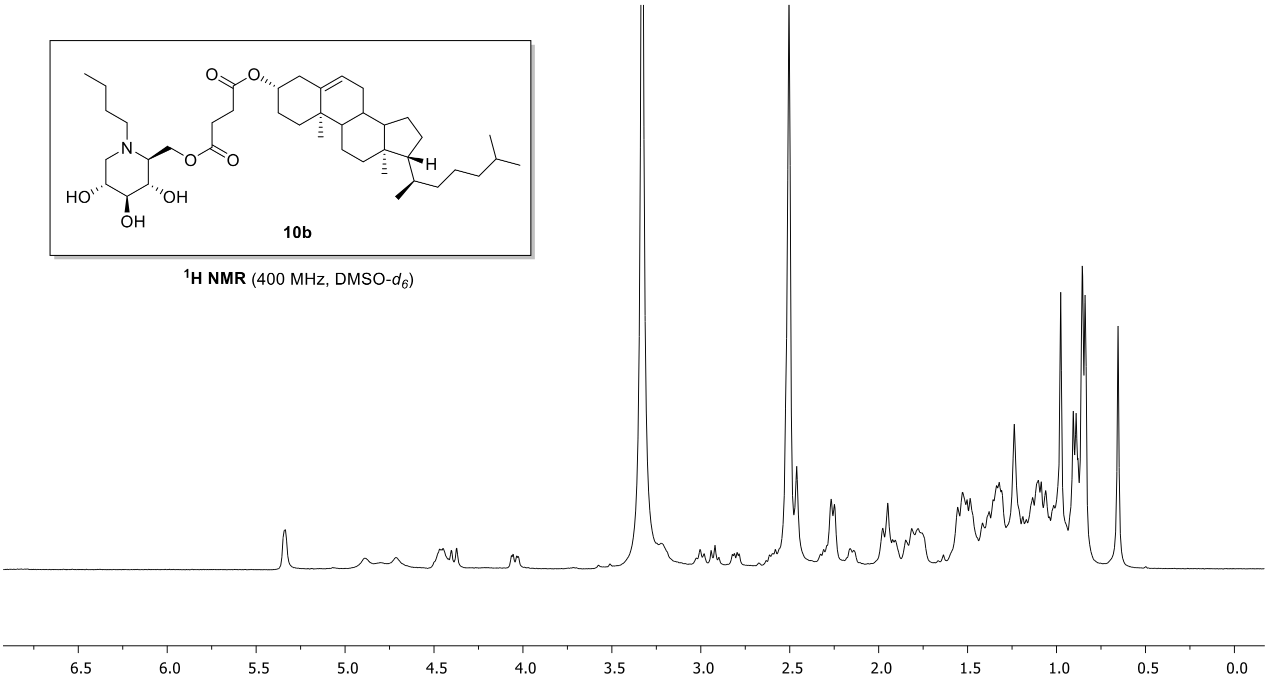

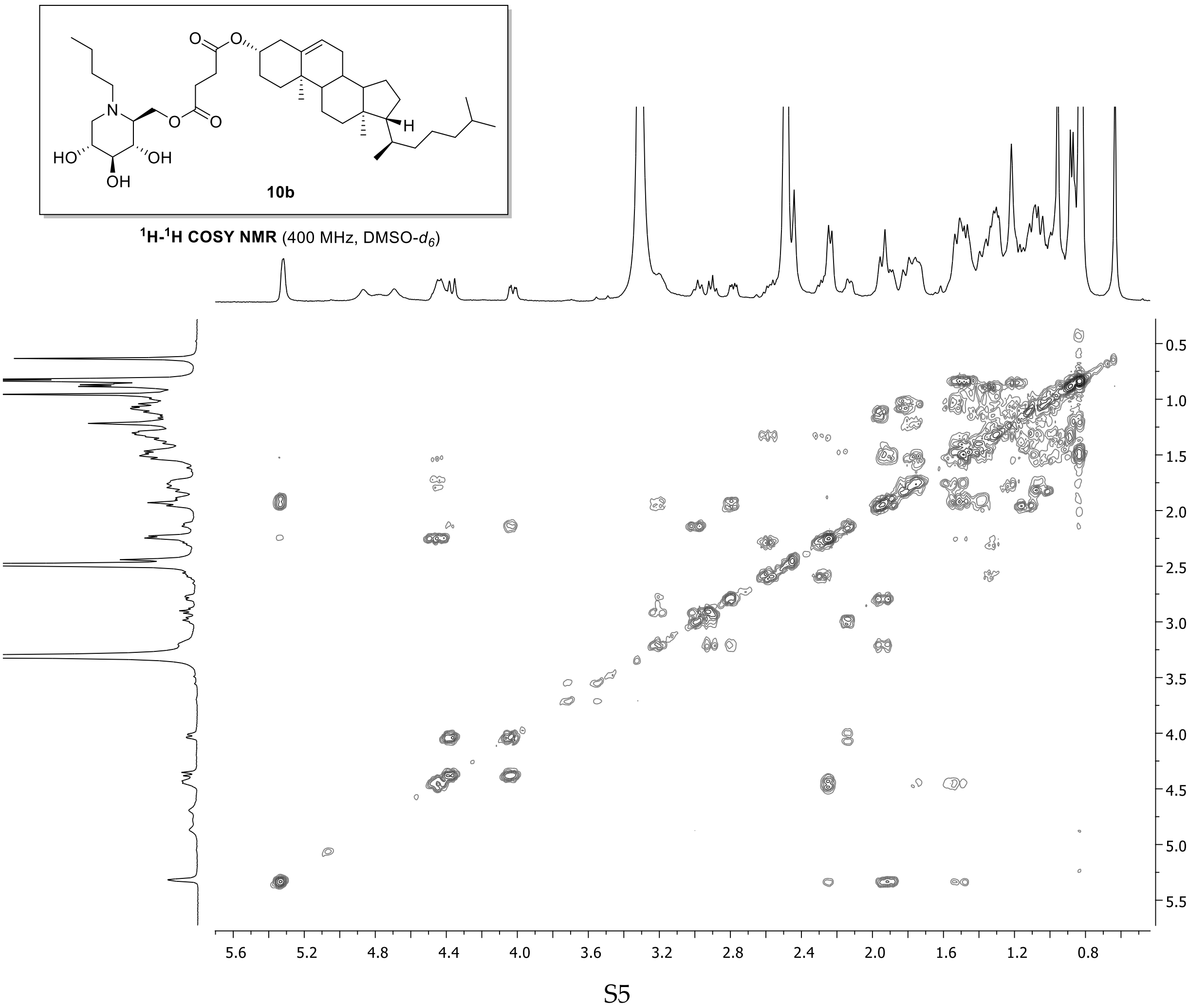

FIGURE S5. ^{1}H and ^{13}C NMR spectra of compound **11a.**

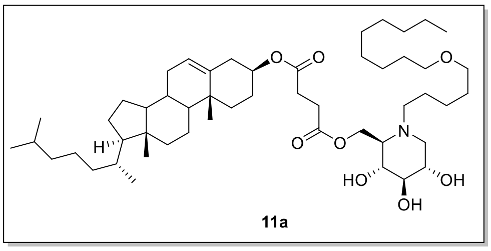

^{1}H NMR (400 MHz, CD_3OD)

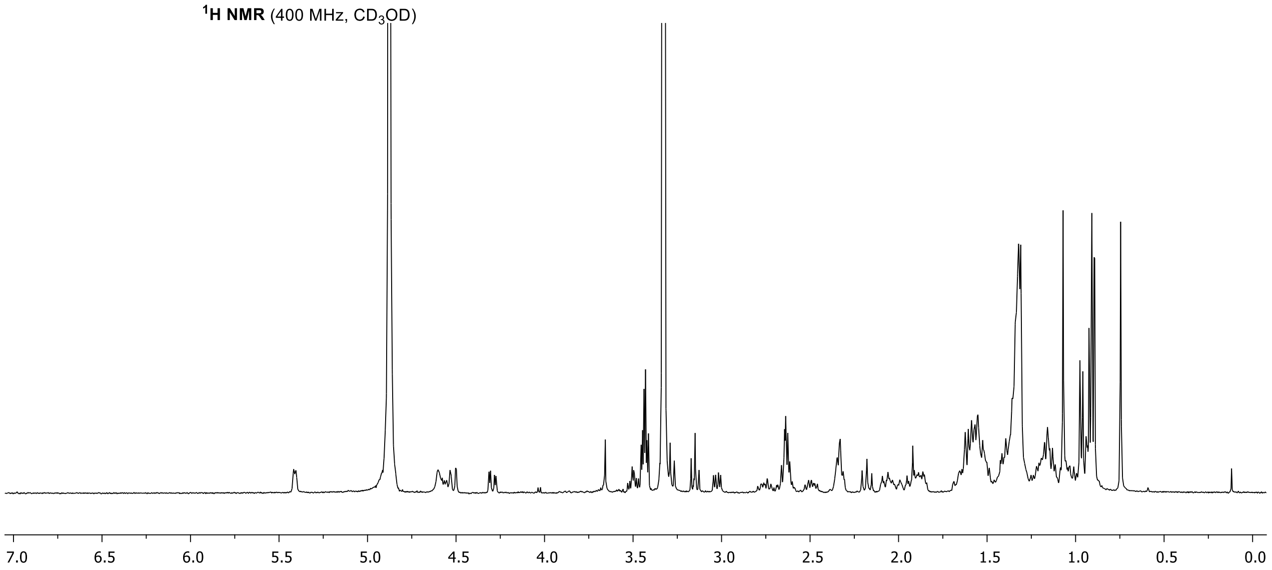

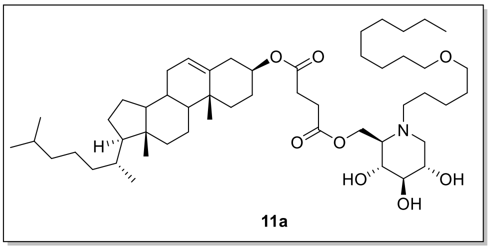

^{13}C NMR (400 MHz, CD_3OD)

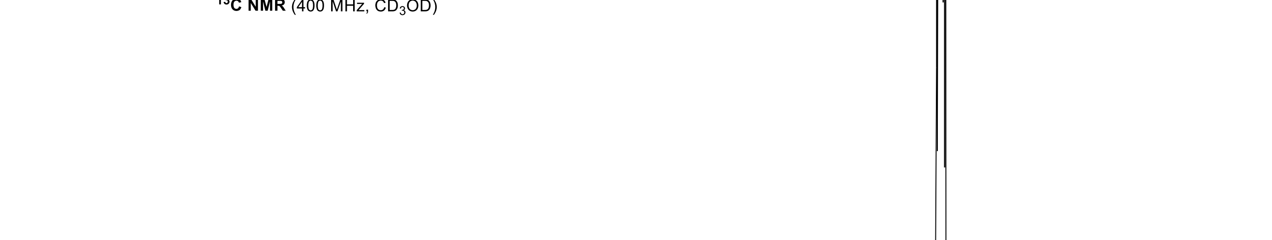

FIGURE S6. ^{1}H and ^{1}H-^{1}H COSY NMR spectra of compound **11b**

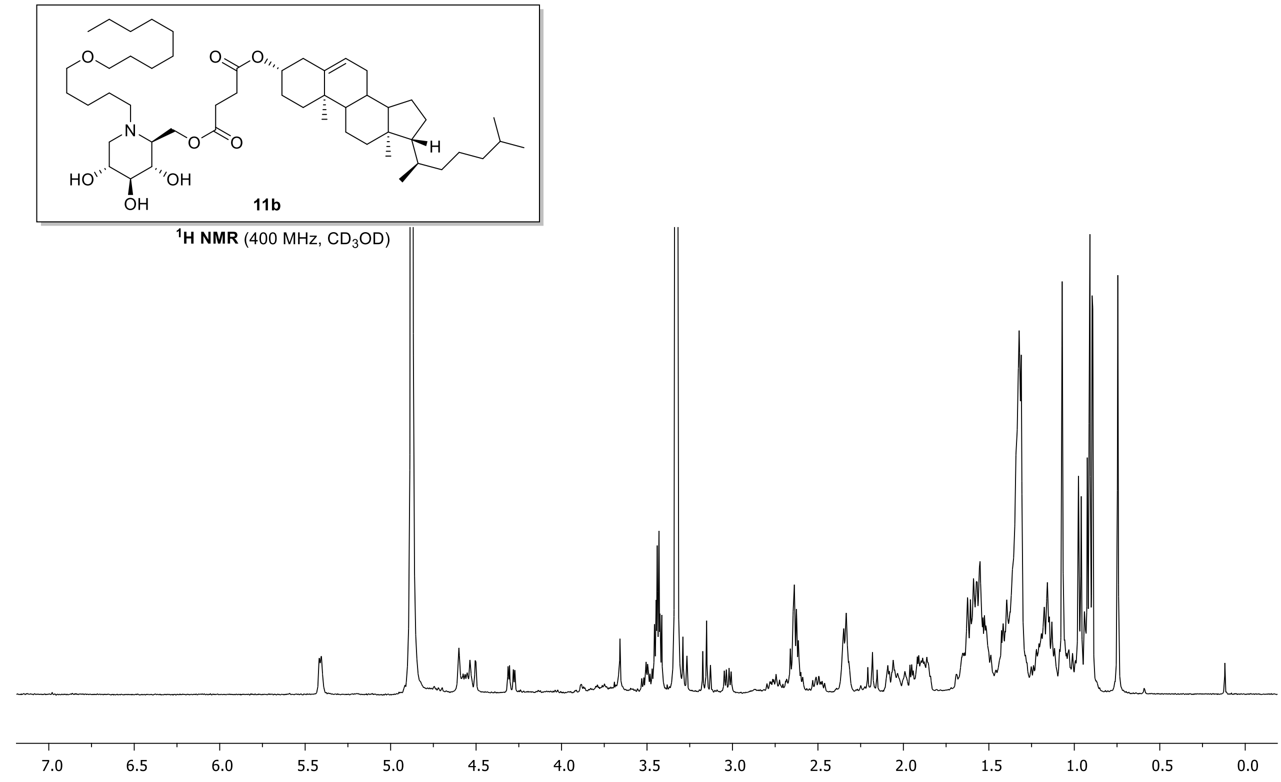

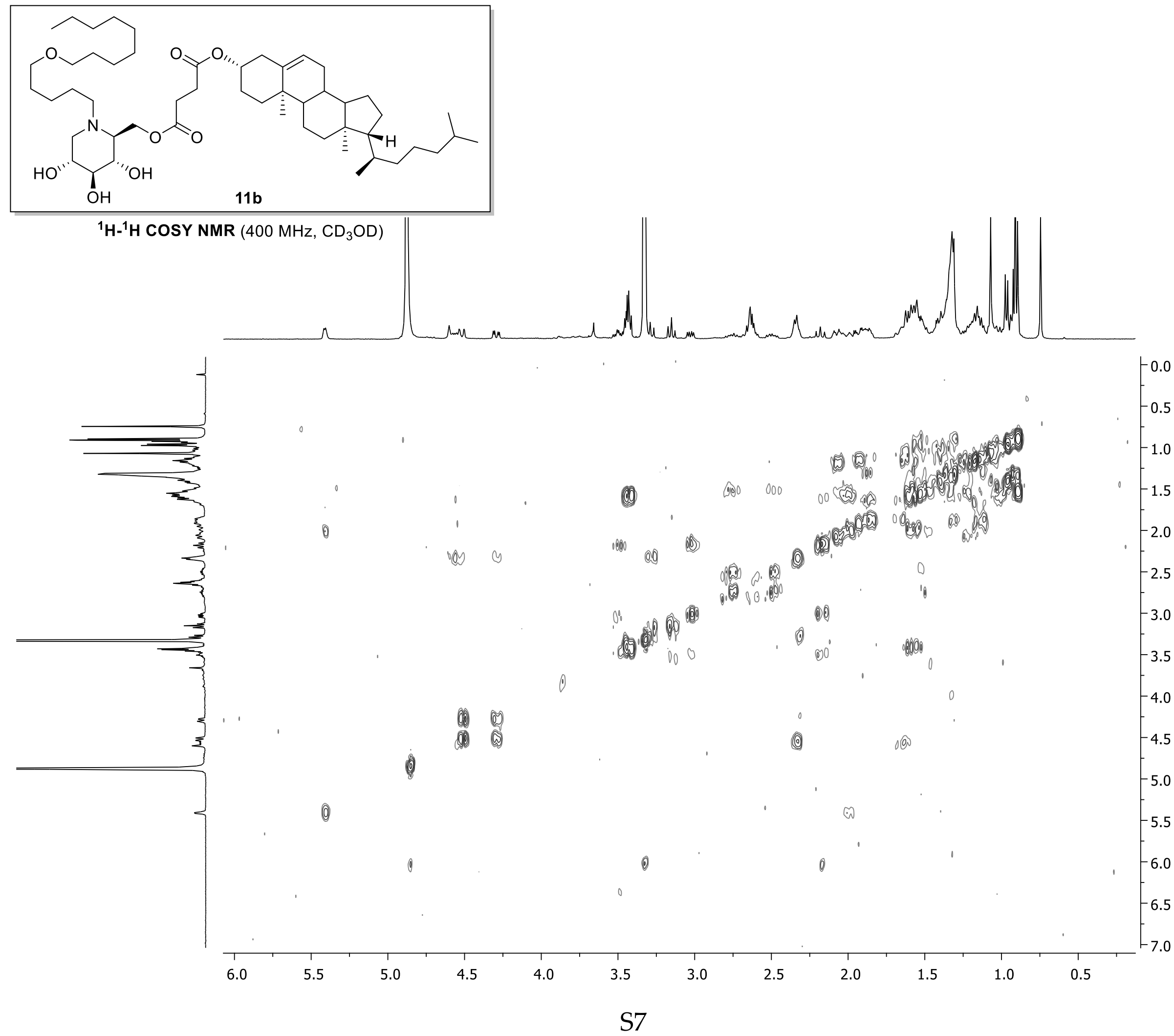

Supplement: Supplementary file 1 [file marinedrugs-18-00572-s001.pdf]
